# Efficacy and safety of standard and low dose ticagrelor versus clopidogrel in east AsianPatients with chronic total occlusion undergoing percutaneous coronary intervention: a single center retrospective study

**DOI:** 10.1186/s12872-019-01307-0

**Published:** 2020-03-05

**Authors:** Yong Wang, Hong-wei Zhao, Cheng-fu Wang, Xiao-jiao Zhang, Jie Tao, Chun-sheng Cui, Qing-kun Meng, Yu Zhu, De-feng Luo, Ai-jie Hou, Bo Luan

**Affiliations:** Department of Cardiology, The People’s Hospital of China Medical University, The People’s Hospital of Liaoning Province, No. 33, Wenyi road, Shenhe District, Shenyang City, Liaoning Province China

**Keywords:** Ticagrelor, Clopidogrel, Chronic total occlusion, Percutaneous coronary intervention

## Abstract

**Background:**

Patients with coronary chronic total occlusion (CTO) require effective antiplatelet therapy after percutaneous coronary intervention (PCI). Ticagrelor has more pronounced platelet inhibition than clopidogrel. However, the most appropriate dose of ticagrelor in East Asian populations remains unclear.

**Method:**

We compared ticagrelor (180 mg loading dose, 90 mg twice daily thereafter and 120 mg loading dose, 60 mg twice daily thereafter) and clopidogrel (300 mg loading dose, 75 mg daily thereafter) for prevention of cardiovascular events in 525patients with CTO undergoing PCI.

**Results:**

The rate of in-hospital major adverse cardiac and cerebral events (MACCE) was not different between the groups. At 1-year follow-up, target vessel revascularization (TVR) in both ticagrelor groups were significantly lower than that in the clopidogrel group (*p* = 0.047); TVR was significantly decreased in 60 mg ticagrelor compared to standard dose clopidogrel (*p* = 0.046). At 1-year follow-up, overall MACCE in both ticagrelor groups were significantly lower than that in the clopidogrel group (*p* = 0.023). Kaplan–Meier analysis showed MACCE-free survival was significantly higher in both ticagrelor groups than in the clopidogrel group (*p* = 0.024). During hospitalization, minor bleeding was significant increased in the 90 mg ticagrelor group (*p* = 0.021). At 1-year follow-up, risk of major and minor bleeding were significantly increased in the 90 mg ticagrelor group.

**Conclusion:**

In East Asian patients with CTO undergoing PCI, 60 mg ticagrelor was as effective as 90 mg, at the same time significantly reduced risk of bleeding.

## Introduction

CTO was defined as thrombolysis in myocardial infarction (TIMI) grade-0 flow with a duration of 3 months, documented angiographically or clinically defined [1]. From previous studies, approximately 20% of patients with coronary heart disease have at least one CTO lesion [1]. Accumulating evidence has confirmed that successful revascularization can effectively improve myocardial ischemia, relieve angina [2], improve left ventricular function [3], and improve clinical outcomes [4, 5] in CTO patients. However, a higher risk of CTO-PCI, a longer duration of the procedure, and a larger number of implanted stents required more intensive anticoagulation during the perioperative period. Ticagrelor is a reversible antagonist of the P2Y12 receptor with a more rapid onset and more pronounced platelet inhibition, it is widely used in dual antiplatelet therapy following PCI. Studies have shown that ticagrelor can significantly reduce the rate of the composite endpoints of cardiovascular death, myocardial infarction, and stroke compared to clopidogrel, and does not cause an increase in major bleeding events [6]. However, most of the patients included in these clinical studies were from European and American Caucasian populations. Currently, an increasing number of studies have confirmed that the risk of thrombosis and hemorrhagic disease in East Asian populations is different from those in Caucasian populations [7, 8]. Therefore, it is important to investigate suitable doses of ticagrelor in East Asian populations.

This study sought to investigate whether low-dose ticagrelor (120 mg loading dose, 60 mg twice daily thereafter) could reduce risk of bleeding events in CTO patients following PCI, meanwhile whether the ischemic events could be increased compared to compared to standard dose of ticagrelor and 75 mg clopidogrel, thereby providing a reference for clinical decision making.

## Method

### Study design and patients

**Fig. 1:**
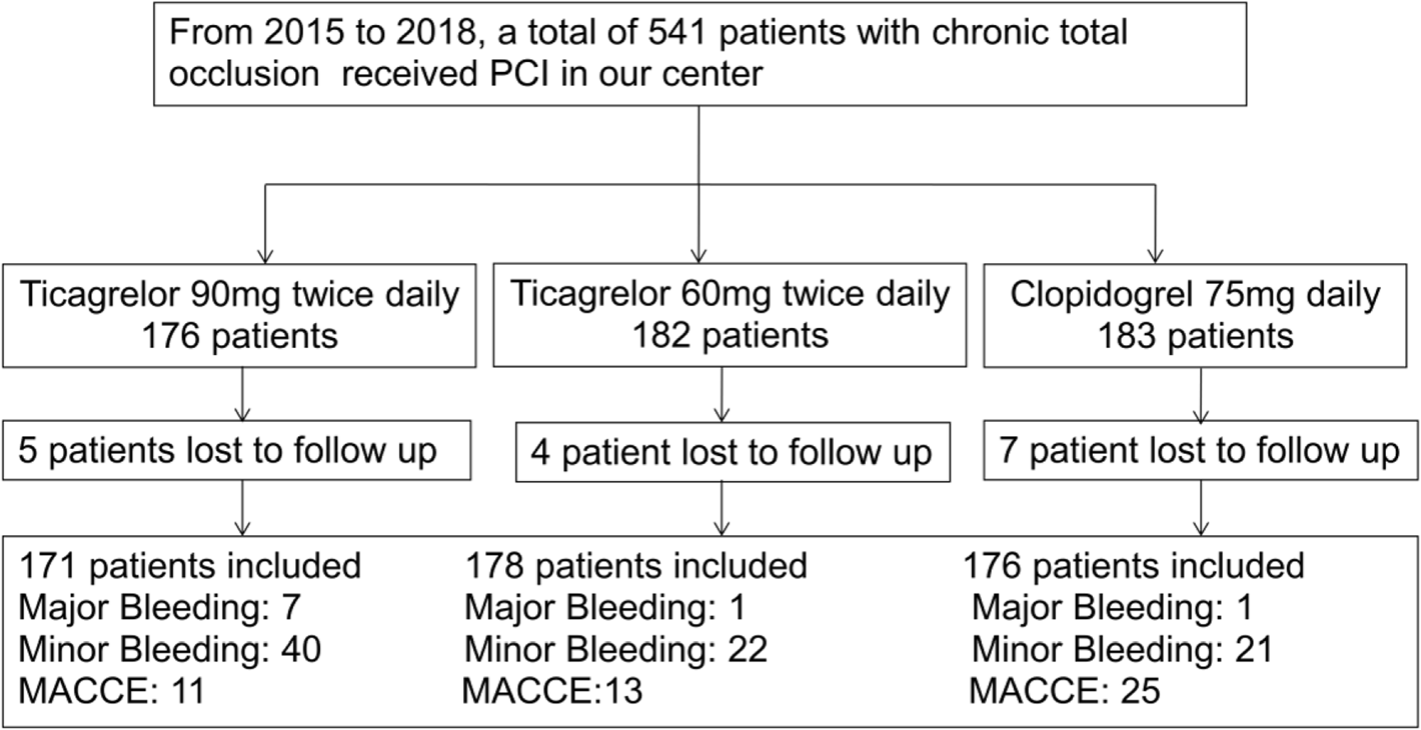
Study profile

In this study, patients with a confirmed diagnosis of CTO undergoing successfully PCI were retrospective selected from January 2015 to May 2018 in our hospital. Exclusion criteria: pregnant women, patients allergic to aspirin, clopidogrel,or ticagrelor; refusal of PCI or failure of stents implantation, patients with an indication of anticoagulants, patients with comorbidities (including coronary artery perforation, intramural hematomas and any comorbidities that may resulted in a worse prognosis), life expectancy less than 1 year, refusal to participate in the trial. A total of 541 patients were enrolled in the study, of which 16 were lost to follow-up, and eventually 525 CTO patients who successfully underwent PCI were included in the study. Of these patients, 171 received a loading dose of 180 mg ticagrelor followed by a maintenance dose of 90 mg twice daily, 178 received a loading dose of 120 mg ticagrelor followed by a maintenance dose of 60 mg twice daily, and 176 received a loading dose of 300 mg clopidogrel followed by 75 mg daily. All patients received a loading dose of 300 mg aspirin followed by a daily oral dose of 100 mg (Fig. 1).

The routine access of the procedure was radial artery, and the physician determined the access as needed. During the procedure, a standard dose of unfractionated heparin (100 IU/kg) was used for anticoagulant therapy, and 2000 IU of unfractionated heparin was added every hour. The use of glycoprotein IIb/IIIa inhibitor (GPI) was left to the physician’s discretion. The study was approved by the Institutional Review Board of the People’s Hospital of Liaoning Province, and all patients signed informed consent before participation.

### Study end points

The primary efficacy endpoint of the study was a composite of adverse cardiovascular events, including all-cause mortality, target vessel revascularization, stent thrombosis, nonfatal myocardial infarction, and nonfatal stroke within one year of follow-up. Myocardial infarction was defined as the presence of pathological Q waves in at least two consecutive leads, or although there was no pathological Q wave, the markers of myocardial injury increased to more than twice the upper limit of normal value, and this increase was not related to PCI or bypass [9]. The primary safety endpoint was defined as complications associated with bleeding during the follow-up period. Bleeding was defined based on the Bleeding Academic Research Consortium criteria [10]. Minor bleeding was defined as skin bruising, subcutaneous ecchymosis, bleeding gums, nosebleed, and so on; major bleeding was defined as gastrointestinal bleeding, intracranial hemorrhage, hemoglobin decrease of ≥3 g/dL, significant bleeding requiring blood transfusion, and fatal bleeding.

### Statistical analysis

Statistical Package for Social Sciences (SPSS) for Windows 20 (IBM SPSS Inc., Chicago, IL) was used for statistical analysis. Normally distributed continuous data are presented as mean ± standard deviation, and those not normally distributed are shown as median (min-max). Categorical variables are shown as numbers and percentages. To compare continuous variables, the Student t-test or Mann-Whitney U test were used, and to compare categorical variables, the chi-square test and the Fisher’s exact test were used in the case of sparse data. Kaplan-Meier graphs were used to access survival without MACCE. All tests were two sided, and *p* values < 0.05 was considered significant.

## Results

In this study, 541 patients with CTO-PCI were recruited consecutively from January 2015 to May 2018 at our hospital. All the procedures were performed by one physician (corresponding author: Bo Luan). All the patients received guideline based optimal medical therapy. The follow-up endpoint was May 2019. 16 patients were lost to follow-up, and in the end 525 CTO patients who successfully underwent coronary interventional therapy were included in the study (Fig. 1). The baseline and procedural characteristics were not statistically different among the three groups and the medication used were comparable between the groups (Tables 1 and 2).

**Table 1:** Clinical characteristics of study population

Variables	Ticagrelor 90 mg (*n* = 171)	Ticagrelor 60 mg (*n* = 178)	Clopidogrel 75 mg (*n* = 176)	*P*-value
Age (years)	63.5 ± 7.4	65.3 ± 8.2	64.9 ± 7.1	0.469
Gender (female), n(%)	62 (36.3%)	68 (38.2%)	67 (40.3%)	0.741
Body mass index (Kg/m^2^)	23.8 ± 3.4	23.5 ± 3.8	24.2 ± 3.9	0.869
Diabetes Mellitus, n(%)	52 (30.4%)	58 (32.6%)	56 (31.8%)	0.910
Hypertension, n(%)	92 (53.8%)	85 (47.8%)	82 (46.6%)	0.363
Current smoker, n(%)	69 (40.4%)	75 (42.1%)	72 (40.9%)	0.937
Previous MI, n(%)	82 (48.0%)	80 (44.9%)	78 (44.3%)	0.772
Previous ischemic CVA, n(%)	39 (22.8%)	45 (25.3%)	38 (21.6%)	0.710
Previous GI bleeding, n(%)	7 (4.1%)	8 (4.5%)	5 (2.8%)	0.716
Peptic Ulcer, n(%)	21 (12.3%)	25 (14.0%)	24 (13.6%)	0.886
LVEF(%)	43.2 ± 5.5	44.4 ± 6.7	43.9 ± 5.8	0.863
NYHA 2–3 on admision, n(%)	115 (67.3%)	109 (61.2%)	112 (63.6%)	0.499
eGFR, ml/min/1.73 mm2	64.5 ± 21.7	65.1 ± 21.9	63.9 ± 22.4	0.782
CRUSADE score	32.1 ± 5.2	33.4 ± 5.5	32.8 ± 6.2	0.792
Medication				
ACEI/ARB, n(%)	80 (46.8%)	75 (42.1%)	82 (46.6%)	0.622
Beta-blockers, n(%)	75 (43.9%)	69 (38.8%)	78 (44.3%)	0.515
Statin, n(%)	165 (96.5%)	170 (95.5%)	166 (94.3%)	0.634
PPI, n(%)	166 (97.1%)	174 (97.8%)	170 (96.6%)	0.807

Abbreviations: MI, myocardial infarction; CVA, cerebrovascular accident; GI, gastrointestinal; LVEF, left ventricular ejection fraction; NYHA, New York Heart Association; eGFR, estimated glomerular filtration rate; ACEIs, angiotensin-converting enzyme inhibitors; ARBs, angiotensin receptor blockers; PPI, proton pump inhibitor

**Table 2:** Procedure-related characteristics of the studied patients

Variables	Ticagrelor 90 mg (*n* = 171)	Ticagrelor 60 mg (*n* = 178)	Clopidogrel 75 mg (*n* = 176)	*P*-value
Site of access				
Radial	58 (33.9%)	52 (29.2%)	54 (30.7%)	0.627
Femoral	23 (13.5%)	41 (23.0%)	35 (19.9%)	0.067
Radial and Femoral	90 (52.6%)	85 (47.8%)	87 (49.4%)	0.657
Total amount of conrrast (ml)	233.8 ± 102.0	228.3 ± 98.6	215.8 ± 94.6	0.327
Total time of procedure (min)	123.6 ± 55.9	119.8.1 ± 57.5	128.4 ± 64.3	0.621
LAD occlusion, n (%)	60 (35.1%)	55 (30.9%)	58 (33.0%)	0.716
LCX occlusion, n (%)	32 (18.7%)	29 (16.3%)	28 (15.9%)	0.766
RCA occlusion, n (%)	89 (52.0%)	96 (53.9%)	92 (52.3%)	0.939
Occlusion lesion length, mm	58.4 ± 26.3	55.9 ± 24.6	56.6 ± 25.4	0.748
Retrograde filling > grade 2	85 (49.7%)	82 (46.1%)	81 (46.0%)	0.730
Reverse wire technique	44 (25.7%)	51 (28.7%)	48 (27.3%)	0.827
IABP, n(%)	12 (7.0%)	9 (5.1%)	11 (6.3%)	0.743
IVUS, n(%)	34 (19.9%)	38 (21.3%)	36 (20.5%)	0.953
Number of stents per patient	2.8 ± 1.9	2.6 ± 1.8	2.7 ± 2.0	0.568
Stent length (mean, mm)	65.9 ± 26.3	66.4 ± 29.5	67.2 ± 31.4	0.382
TIMI III flow after PCI	152 (88.9%)	162 (91.0%)	158 (89.8%)	0.814
Glycoprotein IIb/IIIa receptor inhibitor, n(%)	46 (26.9%)	55 (30.9%)	52 (29.5%)	0.716
LMWH, (%)	50 (29.2%)	48 (27.0%)	56 (31.8%)	0.611

Abbreviations: *LAD* Left anterior descending artery, *LCX* Left circumflex coronary artery, *RCA* Right coronary artery *IABP*, Intra-aortic balloon pump, *IVUS* Intravascular ultrasonography, *TIMI* Thrombolysis in myocardial infarction, *LMWH* Low-molecular-weight heparin

### Efficacy endpoint events

**Fig. 2:**
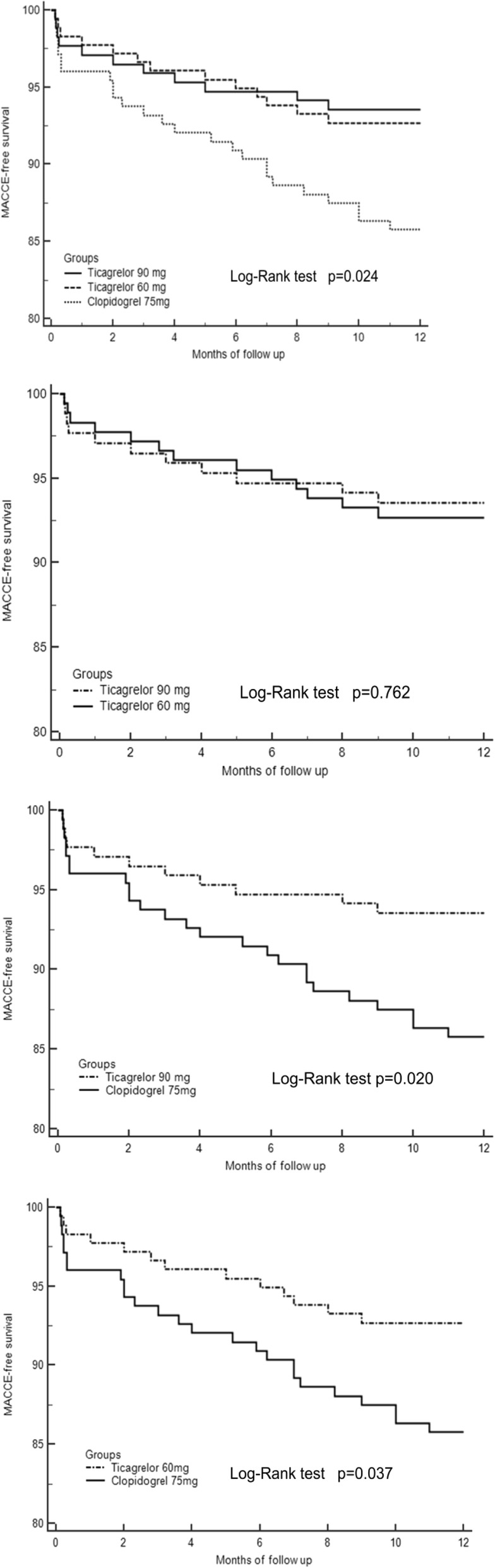
Cumulative MACCE-free survival

There was no difference in the rate of in-hospital major adverse cardiac and cerebral events (MACCE) between the studied groups. However, at 1-year follow-up, target vessel revascularization (TVR) in the two ticagrelor dose groups were significantly lower than that in the clopidogrel group (2.8% vs. 2.3% vs. 7.4%, *p* = 0.047). TVR in 60 mg ticagrelor group was similar with 90 mg grous (2.3% vs. 2.8%, *p* = 1.00) and was significantly lower compared to the standard dose of clopidogrel (2.8% vs. 7.4%, *p* = 0.046). At 1-year follow-up, the overall MACCE of the two ticagrelor dose groups were significantly lower than that of the clopidogrel group (7.3% vs. 6.4% vs. 14.2%, *p* = 0.023). MACCE in 60 mg ticagrelor group was similar with 90 mg ticagrelor groups (7.3%vs. 6.4%, *p* = 0.748) and was significantly decreased compared to the standard dose of clopidogrel (7.3% vs. 14.2%, *p* = 0.036) (Table 3). Kaplan–Meier analysis showed that MACCE-free survival was significantly higher in ticagrelor groups than in the 75 mg clopidogrel group (*p* = 0.024), MACCE-free survival was significantly higher in the 90 mg ticagrelor group compared to the clopidogrel group (*p* = 0.020), MACCE-free survival was significantly higher in the 60 mg ticagrelor group compared to the clopidogrel group (*p* = 0.037), and there was no significant difference between the 60 mg and 90 mg ticagrelor groups (*p* = 0.762) (Fig. 2).

**Table 3:** MACCE of In-Hospital and 12-Months Follow-Up

Variables	Ticagrelor 90 mg (*n* = 171)	Ticagrelor 60 mg (*n* = 178)	Clopidogrel 75 mg (*n* = 176)	*P*-value
MACE in hospital				
All-cause mortality, n(%)	1 (0.6%)	1 (0.6%)	2 (1.1%)	0.848
MI, n(%)	2 (1.2%)	1 (0.6%)	2 (1.1%)	0.749
Stent thrombosis, n(%)	1 (0.6%)	2 (1.1%)	4 (2.3%)	0.465
Stroke, n(%)	1 (0.6%)	1 (0.6%)	1 (0.6%)	1
TVR, n(%)	3 (1.8%)	2 (1.1%)	7 (4.0%)	0.213
Overall MACCE, n(%)	5 (2.9%)	5 (2.8%)	9 (5.1%)	0.454
MACE during 12-month follow-up				
All-cause mortality, n(%)	2 (1.2%)	1 (0.6%)	2 (1.1%)	0.749
MI, n(%)	2 (1.2%)	3 (1.7%)	5 (2.8%)	0.576
Stent thrombosis, n(%)	4 (2.3%)	4 (2.2%)	6 (3.4%)	0.792
Stroke, n(%)	1 (0.6%)	0	1 (0.6%)	0.551
TVR, n(%)	4 (2.3%)	5 (2.8%)	13 (7.4%)	**0.047**
Overall MACCE, n(%)	11 (6.4%)	13 (7.3%)	25 (14.2%)	**0.023**

Abbreviations: *MACCE* Major adverse cardiac and cerebral events, *TVR* Target vessel revascularization, *MI* Myocardial infarction

### Safety endpoint events

During hospitalization, there was a significant increase in the risk of minor bleeding in the 90 mg ticagrelor group (18.1% vs. 10.1% vs. 9.1%, *p* = 0.021), although no increased risk of major bleeding was observed. The risk of minor bleeding in the 60 mg ticagrelor group was significantly lower than in the 90 mg ticagrelor group (10.1% vs. 18.1%, *p* = 0.044) and similar to that of the clopidogrel group (10.1% vs. 9.1%, *p* = 0.857). At the 1-year follow-up, the risk of major bleeding (4.1% vs. 0.6% vs. 0.6%, *p* = 0.016) and minor bleeding (23.4% vs. 12.4% vs. 11.9%, *p* = 0.004) were significantly higher in the 90 mg ticagrelor group. The risk of major bleeding (0.6% vs. 4.1%, *p* = 0.034) and minor bleeding (12.4% vs. 23.4%, *p* = 0.007) were significantly lower in the 60 mg ticagrelor group than in the 90 mg ticagrelor group. There was no significant difference in the risk of major bleeding (0.6% vs. 0.6%, *p* = 1.000) or minor bleeding (12.4% vs. 11.9%, *p* = 1.000) between the 60 mg ticagrelor group and the clopidogrel group (Table 4).

**Table 4:** Bleeding events of In-Hospital and 12-Months Follow-Up

Variables	Ticagrelor 90 mg (*n* = 171)	Ticagrelor 60 mg (*n* = 178)	Clopidogrel 75 mg (*n* = 176)	*P*-value
Bleedings in hospital				
Major bleeding, n(%)	2 (1.2%)	1 (0.6%)	1 (0.6%)	0.699
Minor bleeding, n(%)	31 (18.1%)	18 (10.1%)	16 (9.1%)	**0.021**
Bleedings during 12-month follow-up				
Major bleeding, n(%)	7 (4.1%)	1 (0.6%)	1 (0.6%)	**0.016**
Minor bleeding, n(%)	40 (23.4)	22 (12.4%)	21 (11.9%)	**0.004**

## Discussion

This is the first study to investigate the efficacy and safety of a low-dose ticagrelor (60 mg twice daily) antiplatelet regimen in East Asian patients with CTO undergoing PCI. We found that low-dose ticagrelor achieved similar effect in reducing the risk of revascularization and overall MACCE compared to 90 mg group with a significant lower risk of major and minor bleeding events.

A higher risk of CTO-PCI, a longer duration of the procedure, and a larger number of implanted stents required more intensive anticoagulation during the perioperative period. However, the optimal antiplatelet therapy after CTO-PCI is still unclear. In 2014 we found that 90 mg ticagrelor for CTO-PCI resulted in a significant bleeding events in asian patients and 75 mg clopidogrel resulted in a significant ischemic events, at that time, we assumed that a low dose ticagrelor may be an alternative. So we start our study in 2015. Ticagrelor 60 mg tablet was on market in China since March 2018. Before that, we use 2/3 of 90 mg, after March 2018, 60 mg is available. In this study, we retrospectively analyse a low dose of ticagrelor (120 mg loading dose, 60 mg twice daily thereafter) for prevention of cardiovascular events in 525 patients with CTO undergoing PCI.

A growing body of evidence has shown that ticagrelor can significantly reduce the rate of the composite end points of cardiovascular death, myocardial infarction, and stroke compared to clopidogrel, and does not cause an increase in major bleeding events [6]. However, most of the patients included in these clinical studies were from European and American Caucasian populations. Currently, an increasing number of studies have confirmed that the risk of thrombosis and hemorrhagic disease in East Asian populations is different from those in Caucasian populations [7, 8]. During antithrombotic treatment, East Asian patients tended to have a lower risk of ischemic events especially in coronary artery disease and a higher incidence of bleeding [7, 8]. Some researchers suggested that East Asian patients have a lower risk of ischemic events after PCI despite a lower response to clopidogrel [11–13]. In addition, studies have shown that East Asian populations have significantly increased risk of gastrointestinal bleeding even with low-intensity antiplatelet therapy (such as low-dose aspirin) compared to Caucasians [14]. Therefore, it is important to investigate suitable doses of ticagrelor in East Asian populations.

The GEMINI-ACS-1 randomized study confirmed that Asia Pacific patients with acute coronary syndrome had significantly more clinically significant bleeding events during antithrombotic therapy compared to other regions [15]. A study from South Korea showed that patients prescribed 90 mg ticagrelor had a 40.6% risk of bleeding in one month follow up [16]. Studies have confirmed that the presence of ticagrelor and its major active metabolite (ARC124910XX) is significantly higher in East Asian population than in the Caucasian population [17, 18]. Therefore, a low dose of ticagrelor in the East Asian population can achieve a similar rate of platelet inhibition while reducing the risks of bleeding. In the sub-study of the PEGASUS-TIMI 54 study, post-dose plasma level of ticagrelor was 38% lower with 60 mg than with 90 mg, but levels of platelet reactivity in both doses did not differ [19]. Similarly, low-dose ticagrelor (60 mg twice daily) and standard-dose ticagrelor (90 mg twice daily) exhibited similar 30-day rates of platelet inhibition in East Asian ACS patients [20].

A crossover study including Chinese patients with stable coronary artery disease evaluated the antiplatelet effect of very low-dose of ticagrelor (22.5 mg twice daily) vs. standard-dose of clopidogrel (75 mg daily) for 7 days. They found that the level of platelet reactivity in the ticagrelor group was significantly lower than that in the clopidogrel group [21]. Thus, we have reason to believe that low doses of ticagrelor are more suitable for the Asian population. We sought to investigate the efficacy and safety of low-dose (60 mg) ticagrelor in antiplatelet therapy of CTO patients following PCI. Similar to previous studies, low-dose ticagrelor could significantly reduce composite MACCE compared to standard dose of clopidogrel. In addition, the associated risks of bleeding, including major and minor bleeding, were significantly reduced compared to the 90 mg dose. To some extent, this indicates that low doses of ticagrelor are effective and safe in the Asian population.

In clinical practice, it is of vital inportanct to assess the ischemic and bleeding risk for the individual, since antiplatelet therapy plays pivotal role in patients with CTO-PCI. The GRACE and CRUSADE score are widely recommended for the ischemic events and bleeding risk classification, respectively. For patients at high risk of bleeding, we speculate that low-dose ticagrelor is superior to standard-dose clopidogrel. In this study, subgroup analysis was not performed due to sample size limitations, which may lead to some bias in the results.

Although this study investigated the efficacy and safety of low-dose ticagrelor antiplatelet therapy following successful PCI in East Asian CTO patients, the following limitations exist: (1) The present study did not conduct measurements of cytochrome P450 2C19 gene, which is critical for clopidogrel metabolism; (2) The present study is a single-center retrospective analysis with a small sample size, which may lead to research bias, (3) Because of the small sample size, no subgroup analysis was performed. Thus, a large-sample, multi-center, prospective randomized controlled trial is needed in the future to validate our conclusions.

## Conclusion

In conclusion, our data suggest that In East Asian patients with CTO undergoing PCI, 60 mg ticagrelor was as effective as 90 mg, at the same time significantly reduced risk of bleeding.

## Data Availability

The datasets generated and analysed during the current study are not publicly available due to a further study of this area but are available from the corresponding author on reasonable request.

## Abbreviations

CTO: Coronary chronic total occlusion
GPI: Glycoprotein IIb/IIIa inhibitor
MACCE: Major adverse cardiac and cerebral events
PCI: Percutaneous coronary intervention
TIMI: Thrombolysis in myocardial infarction
TVR: Target vessel revascularization
